# Scalable Production of a Multifunctional Protein (TSG-6) That Aggregates with Itself and the CHO Cells That Synthesize It

**DOI:** 10.1371/journal.pone.0147553

**Published:** 2016-01-21

**Authors:** Dong-Ki Kim, Hosoon Choi, Hidetaka Nishida, Joo Youn Oh, Carl Gregory, Ryang Hwa Lee, Ji Min Yu, Jun Watanabe, Su Yeon An, Thomas J. Bartosh, Darwin J. Prockop

**Affiliations:** Institute for Regenerative Medicine, Texas A&M Health Science Center, College of Medicine at Scott and White, Temple, Texas, United States of America; Instituto Butantan, BRAZIL

## Abstract

TNF-α stimulated gene/protein 6 (TNFAIP6/TSG-6) is a multifunctional protein that has a number of potential therapeutic applications. Experiments and clinical trials with TSG-6, however, have been limited by the technical difficulties of producing the recombinant protein. We prepared stable clones of CHO cells that expressed recombinant human TSG-6 (rhTSG-6) as a secreted glycoprotein. Paradoxically, both cell number and protein production decreased dramatically when the clones were expanded. The decreases occurred because the protein aggregated the synthesizing CHO cells by binding to the brush border of hyaluronan that is found around many cultured cells. In addition, the rhTSG-6 readily self-aggregated. To address these problems, we added to the medium an inhibitor of hyaluronan synthesis and heparin to compete with the binding of TSG-6 to hyaluronan. Also, we optimized the composition of the culture medium, and transferred the CHO cells from a spinner culture system to a bioreactor that controlled pH and thereby decreased pH-dependent binding properties of the protein. With these and other improvements in the culture conditions, we obtained 57.0 mg ± 9.16 S.D. of rhTSG-6 in 5 or 6 liter of medium. The rhTSG-6 accounted for 18.0% ± 3.76 S.D. of the total protein in the medium. We then purified the protein with a Ni-chelate column that bound the His tag engineered into the C-terminus of the protein followed by an anion exchange column. The yield of the purified monomeric rhTSG-6 was 4.1 mg to 5.6 mg per liter of culture medium. After intravenous injection into mice, the protein had a longer plasma half-life than commercially available rhTSG-6 isolated from a mammalian cell lysate, apparently because it was recovered as a secreted glycoprotein. The bioactivity of the rhTSG-6 in suppressing inflammation was demonstrated in a murine model.

## Introduction

TNF-α stimulated gene/protein 6 (TNFAIP6/TSG-6) is a multifunctional endogenous protein that is expressed by a variety of cells in response to stimulation by pro-inflammatory cytokines [1–5]. The protein is 35 kDa and consists primarily of a N-terminal link domain similar to the hyaluronan-binding module of proteoglycans, and a C-terminal domain with sequences similar to complement C1r/C1s, an embryonic sea urchin growth factor Uegf and BMP1 (CUB domain) [6, 7]. TSG-6 binds to a large number of components of the extracellular matrix including hyaluronan, heparin, heparan sulfate, thrombospondins-1 and -2, fibronectin, and pentraxin 3 [6–10]. These interactions primarily act to stabilize or remodel the extracellular matrix.

In addition, TSG-6 modulates inflammatory responses by several effects, some of which are related to its stabilization of extracellular matrix but some of which appear to be independent. One of the more complex interactions is that the protein catalytically transfers the heavy chains of inter-α-trypsin inhibitor onto hyaluronan [11]. It thereby helps stabilize the extracellular matrix, e.g. during cumulus expansion prior to ovulation [12–15]. Simultaneously, it releases the bikunin component from inter-α-trypsin inhibitor to increase its activity in inhibiting kallikrein during the inflammatory responses [10, 16]. In apparently independent actions, TSG-6 reduces the migration of neutrophils through endothelial cells [17–19], forms a ternary complex with murine mast cell trypases and heparin [20], and inhibits FGF-2 induced angiogenesis through an interaction with pentraxin 3 [21]. In addition, TSG-6 either directly or through a complex with hyaluronan, binds to CD44 on resident macrophages in a manner that decreases TLR2/NF-κB signaling and modulates the initial phase of the inflammatory response of most tissues [22–24]. TSG-6 thereby reduces the large, second phase of inflammation that is frequently an excessive and deleterious response to sterile injuries [25].

These and related observations stimulated interest in the therapeutic potentials of the TSG-6. For example, transgenic mice with localized over-expression of the gene in joints or cartilage had a decreased response to experimentally-induced arthritis [26, 27]. Conversely, mice with a knock-out of the gene had increased susceptibility to proteoglycan-induced arthritis [3]. Also, administration of recombinant TSG-6 decreased experimentally-induced arthritis in several different models [28, 29]. In addition, the recombinant protein decreased osteoblastogenesis and osteoclast activity [30, 31]. Interest in the therapeutic potentials of the protein was further increased by the recent observations that enhanced expression of the protein by adult stem/progenitor cells referred to as mesenchymal stem/stromal cells (MSCs) explained some of the beneficial effects observed after administration of the cells in animal models for myocardial infarction [32], chemical injury to the cornea [22, 23], zymosan-induced peritonitis [24], and LPS-induced or bleomycin-induced lung injury [3, 33, 34].

Experiments with TSG-6 have been limited by the difficulty in producing the recombinant protein in high yields. Recombinant TSG-6 (rhTSG-6) was previously synthesized in insect cells [29, 35], in Chinese hamster ovary cells [28], and in mouse myeloma cells (proprietary protocol; R&D Systems; http://www.rndsystems.com). However, large scale production has not been achieved. The problem was in part addressed by using *E*. *coli* to synthesize the N-terminal portion of the protein that contains the hyaluronan-binding link module [36], and that retains some of the biological properties of the intact protein, including inhibition of neutrophil migration, binding to CXCL8, binding to thrombospondin-1 and enhancement of inter-α-inhibitor/bikunin’s anti-plasmin activity [7, 10, 18, 19].

We have recently developed clones of stably transfected Chinese hamster ovary cells (CHO) that secrete rhTSG-6. We then developed conditions to overcome two major challenges in developing scalable production of the protein: the tendency of rhTSG-6 to aggregate the cells that synthesize it by cross-linking the brush border of hyaluronan that surrounds many cultured cells, and its tendency to self-aggregate in an apparently irreversible manner. We then purified rhTSG-6 from the medium of the transfected CHO cells with two chromatographic steps to achieve a yield of monomeric rhTSG-6 of about 4.1 to 5.6 mg per liter of culture medium.

## Materials and Methods

### Ethical Statement

All the animal experiments were approved by the Institutional Animal Care and Use Committee of Scott & White Memorial Hospital (2014–019, Bioassay for efficacy of adult stem cells and their products). All animals were euthanized with ketamine/xylazine or 5% isoflurane/oxygen and cervical dislocation.

### MSCs culture

Frozen vials of hMSCs from bone marrow (Donor 7302R) were obtained from the Center for the Preparation and Distribution of Adult Stem Cells (formerly http://www.som.tulane.edu/gene_therapy/distribute.shtmLl; currently http://medicine.tamhsc.edu/irm/msc-distribution.html) that supplies standardized preparations of MSCs enriched for early progenitor cells to over 250 laboratories under the auspices of an NIH/NCRR grant (P40 RR 17447).

A frozen vial of about 10^6^ passage 1 hMSCs was thawed, and plated at 200 to 500 cells/cm^2^ in 150 mm diameter plates with 30 ml of complete culture medium (CCM) that consisted of α-minimal essential medium (α-MEM; Invitrogen, Carlsbad, CA), 17% fetal bovine serum (FBS; lot-selected for rapid growth of hMSCs; Atlanta Biologicals, Inc, Norcross, GA), 100 units/ml penicillin, 100 μg/ml streptomycin, and 2 mM L-glutamine (Invitrogen). The cultures were incubated with replacement of medium every 2 days for 7 to 9 days until they were 70% confluent. The cultures were washed with PBS and the MSCs (passage 2) were harvested by incubation for 5 to 10 min at 37°C with 0.25% trypsin and 1 mM EDTA.

### Plasmid construction

Total RNA was isolated from hMSC cells that were stimulated to express TSG-6 by incubation of 3 × 10^4^ cells/cm^2^ over night with 10 ng/ml of TNF-α in CCM containing a reduced concentration of 2% FBS [32]. About 1 μg of total RNA was used to produce the first strand cDNA pool by RT-PCR (Superscript II/oligo dT_12-18_; Invitrogen) using a thermocycler (C100TM Thermal Cycler; BIO-RAD, Hercules, CA). cDNAs encoding hTSG-6 (GenBank accession number: NM_007115) were amplified by PCR using the following primers: 5’- CGGGGTACCATGATCATCTTAATTTACTT -3’ (sense), 5’- GGTGATCAGTGGCTAAATCTTCCA -3’ (anti-sense). The PCR products were sub-cloned into the Kpn I and Spe I sites in multi-cloning sites of a pEF4-Myc/His plasmid (cat, # V942-20; Invitrogen) and the plasmid was amplified in *E*.*coli* DH5α cells (cat. # 18265–017; Invitrogen).

### Synthesis of rhTSG-6-WT in stably transfected CHO cells

Chinese hamster ovary (CHO)-S cells (Invitrogen) were plated at 1 × 10^5^ cells in a 100 mm diameter culture dish with 10 ml IMDM (Iscove’s Modified Dulbecco’s Medium; cat # 12440–053; Thermo Scientific, Carlsbad, CA) containing 5% FBS (Premium Select; cat. # S11550; Atlantic Biologicals, Norcross, GA), 50 unit/ml of penicillin, and 50 μg/ml of streptomycin. After incubation for 2 days, cells were transfected with 30 μg of the plasmid construct for expression of rhTSG-6-WT using 60 μl of lipofectamine reagent (Lipofectamine 2000^TM^; Invitrogen) in serum-reduced medium (Opti-MEM; Invitrogen). Four hours later, the medium was replaced with 10 ml of 5% FBS/ IMDM and the cells further incubated for one day. The following day, the transfected cells were lifted and re-seeded in a 100 mm diameter culture dish with 9 ml of a methylcellulose based medium (ClonaCell-TCS medium, cat. # 03814; Stem Cell Technologies, Vancouver, Canada); http://www.stemcell.com/en/Products/Area-of-Interest/Semi-solid-cloning/ClonaCellHY-Medium-D-without-HAT.aspx) containing 500 μg/ml of Zeocin (Invitrogen) to select transformed clones (Stem Cell Technologies). The samples were cultured 10–14 days to allow the clones to form spheres in the semi-solid selection media. The spheres were isolated using a pipette under a microscope. The spheres were expanded on 48 well plates in 5% FBS/ IMDM containing 500 μg/ml of Zeocin until 70% confluence was achieved. At this point, we tested expression in the cells using immunocytochemistry with fluorescently labeled hTSG-6 antibodies (below). The secretion of proteins from each clone was tested by ELISA and selected clones were further tested by Western blotting of media. The most productive clones were further expanded in 24 well plates and then in 100 mm diameter culture dishes to achieve an adequate number of cells for storage in liquid nitrogen (S1 Fig). After expansion, the clones were sequentially adapted to CD-CHO media.

### ELISA

For ELISA of secreted recombinant protein, a 96-well plate (Maxisorp; Thermo Scientific Nunc, Rochester, NY) was coated overnight at 4°C with 100 μl /each well of 10 μg/ml monoclonal antibody specific for rhTSG-6 [clone A38.1.20 [37]; Santa Cruz Biotechnology, Inc., Dallas, TX] in 0.2 M sodium bicarbonate buffer (pH 9.4). The plates were washed with PBS twice and blocked with 0.25% (wt/vol) BSA and 0.05% (vol/vol) Tween 20 in PBS for 30 min at room temperature. Plates were again washed with PBS. Samples of medium (50 μl) or standards of recombinant human TSG-6 protein (R&D Systems) in blocking buffer were added. After 2 h at room temperature, wells were washed with PBS followed by 50 μl/well of 0.5 μg/ml biotinylated anti-human TSG-6 (TSG-6 Biotinylated PAb Detection Antibody; R&D Systems, Minneapolis, MN). After 2 h, plates were washed with PBS. Fifty microliters streptavidin-HRP (R&D Systems) was added to each well. The plates were covered and incubated for 20 min at room temperature. The plates were washed with PBS, 100 μl substrate solutions (R&D Systems) were added, and the samples were incubated for 10 min at room temperature. Absorbance was read at 450 nm (Fluostar Optima; BMG Labtechnologies, Cary, NC).

### Western blots

Ten μl of each sample were separated by 12% SDS–polyacrylamide gel electrophoresis and blotted onto a polyvinylidene fluoride membrane (PVDF). The blot was incubated with primary antibody TSG-6 (200 μg/ml; clone A38.1.20; Santa Cruz Biotechnology, Inc.) for 1 h at RT, and then with secondary antibody conjugated with horseradish peroxidase (1:4000, Santa Cruz) for 30 min at RT. The gels were visualized with the ECL kit (Amersham Pharmacia, Piscataway, NJ).

### Immunocytochemistry

Cells at about 1×10^4^ cells/cm^2^ were cultured on cover slips (12 mm diameter; cat. # 12-545-82; Fisher Scientific) in 24 well plates (cat. # 3524; Corning Life Sciences, Corning, NY) in 5% FBS/IMDM for 3 days. The samples were fixed for 10 min in 4% paraformaldehyde (PFA), and washed with 1× PBS. The slides were blocked in 1× PBS containing 3% BSA and 0.2% Triton X-100 for 1 h at RT. Cells were then incubated for 1 h at RT in primary antibodies (200 μg/ml; TSG-6 clone A38.1.20; Santa Cruz Biotechnology, Inc. and 400 μg/ml; His(C-Term); Invitrogen) and washed three times with PBS. Cells were incubated with fluorescence-labeled secondary antibodies and nuclei were counter-stained with DAPI (10 μg/ml; Molecular Probes) for 30 min at RT.

To stain aggregates, the aggregates were collected with a cell lifter, transferred to a 50 ml conical tube, washed twice with PBS, and fixed with 4% PFA in PBS for 10 min at room temperature. Then 1 ml of OCT solution (Sakura Finetek) was added to the cell aggregates and they were transferred into a histology mold. The mold was frozen in isopentane (Sigma) chilled by liquid nitrogen. Cryosections (~ 10 μm) were prepared with Microm HM560 cryostat. After blocking with 3% BSA and 0.2% Triton X-100 in 1× PBS for 1 h at RT, the samples were incubated overnight at 4°C in primary antibodies (200 μg/ml; TSG-6 clone A38.1.20; Santa Cruz Biotechnology, Inc. and 10 μg/ml; HABP; amsbio, Cambridge, MA) and washed three times with PBS. The aggregates were incubated with fluorescence-labeled secondary antibodies and observed under a fluorescence microscope (Nikon ECLIPSE 80i; Nikon Instruments Inc., Melville, NY) and digitized with a CCD camera. Images were optimized using Adobe Photoshop 7.0.

### Experiments in spinner cultures to optimize the medium

To optimize culture conditions, individual clones of transfected cells were each plated in two 150 mm diameter dishes at 3,000 cells/cm^2^ in 30 ml of 5% FBS/IMDM containing 100 μg/ml of Zeocin^TM^ selection reagent (cat. # R250-1; Invitrogen). After 2 days, the cells were washed with PBS, lifted with 0.25% trypsin, and suspended in spinner culture bottles (Wheaton, Millville, NJ) at 6 × 10^4^ cells/ml in 500 ml of chemically-defined and protein-free medium (CD CHO Medium, cat. # 10743–029; Invitrogen) without Zeocin. Trial experiments were carried out to test the effects of addition of a series of supplements by adding the following to the medium either separately or in combinations: (a) 11 mM of D-(+)-glucose (Sigma; G6152-100G); (b) 10 ml/l non-essential amino acids (MEM 100 ×; cat. # 11140–050; Invitrogen); (c) 10 ml/l vitamin solution (MEM Vitamin Mixture 100 ×; cat. # 11120–052; Invitrogen); (d) 10 ml/l lipid concentrate (Chemically defined, cat. # 11905–031; Invitrogen); and (e) 10 ml/l of surfactant co-polymer (Pluronic F-68, cat. # 24040–032; Invitrogen). The results (S2 Fig) made it possible to define an optimized chemically-defined and protein-free (OCDPF) medium for subsequent experiments (Table 1).

**Table 1:** Composition of optimized chemically defined protein free medium (OCDPF medium).

Supplements added to 970 ml CD-CHO		Company (cat. #)
HT Supplement*	10 ml	Invitrogen (11067–030)
D-(+)-glucose	2 g	Sigma (G6152)
MEM non-essential amino acids	10 ml	Invitrogen (11140–050)
MEM vitamin solution	10 ml	Invitrogen (11120–052)
1 M Methylumbelliferone sodium salt	50 μl	Sigma (M1508)
Heparin sodium salt	250 mg	Sigma (H4784)

* A Mixture of hypoxanthine (10 mM) and thymidine (1.6 mM).

### Preparation CHO clones for culture in spinner cultures

The medium for the experiments was prepared with 1 liter CHO medium (CD-CHO cat. # 10743–011; Invitrogen) that was supplemented with 10 ml hypoxanthine/thymidine supplement (HT 100 ×, cat. # 11067–030; Invitrogen), 40 ml L-glutamine solution (final concentration 8 mM; L-Glutamine 200 mM; cat. # G6152-100G; Sigma), 2 grams D-(+)-glucose (cat. # G6152-100G; Sigma), 10 ml non-essential amino acids (cat. # 11140–050; Invitrogen), 10 ml MEM vitamin solution (cat. # 11120–052; Invitrogen). In addition, 4-methylumbelliferone sodium salt (M1508; Sigma) was added at 50 μM to decrease hyaluronan synthesis [38]. Also, heparin (H4784, Sigma) was added to a concentration of 250 μg/ml to promote suspension adaptation of TSG-6 stable cell lines and to increase the recovery rate of rhTSG-6 proteins [39].

Clones were each plated in two 150 mm diameter dishes at 3,000 cells/cm^2^ in OCDPF containing 100 μg/ml of Zeocin^TM^ selection reagent. After 2 days, cells were washed with PBS and then were incubated in fresh OCDPF media for an additional day. The cells were washed with PBS and lifted using trypsin. After centrifugation, the cells were re-seeded at about 6 × 10^4^/ml in 1 liter of OCDPF medium for suspension culture in a spinner bottle and further cultured for 3 days.

### Bioreactor culture of stable cell line

For production of TSG-6 in a bioreactor, the cells that had been expanded in spinner bottle for 3 days were suspended at 1 × 10^5^/ml in 5 liter of OCDPF medium, and incubated in a bioreactor (PILOT PLANT SYSTEM, 10 liter capacity, W350040-A Wheaton Science Products, Millville, NJ) that automatically titered the pH to 7.4 and monitored the oxygen content of the medium to 20%.

### Purification of secreted rhTSG-6

The culture medium was clarified by centrifugation at 17,000 × g for 10 min. After brief sonication, the medium was passed through a 0.45 μm filter. The proteins were purified by a histidine binding nickel chelate column. In brief, the medium (5 or 6 liter) adjusted to the pH of the equilibration buffer was loaded on the column (70 ml resin bed; Ni-Sepharose Excel; GE Healthcare Bio-Sciences, Uppsala, Sweden) that had been equilibrated with the binding buffer (500 mM NaCl, 0.1% Triton X-100 and 2 M Urea in 20 mM phosphate buffer at pH 7.4). The column was first washed with 25 column volumes of an endotoxin removal buffer (500 mM NaCl, 0.025% Triton X-114, 0.1% Tween 20, 2 M Urea and 20 mM imidazole in 20 mM phosphate buffer at pH 7.4) [40]. In order to wash out any residual Trion-X114 and Tween 20, the column was washed with 40 column volumes of wash buffer (500 mM NaCl, 2 M Urea, and 10 mM imidazole in 20 mM phosphate buffer at pH 7.4) overnight. The recombinant protein was then recovered with about 400 ml of elution buffer (500 mM NaCl, 2 M Urea, and 500 mM imidazole in 20 mM phosphate buffer at pH 7.4) at a flow rate of about 3.5 ml/min. Fractions of 8 ml were collected and assayed by SDS-PAGE gel electrophoresis and TSG-6 ELISA. Thirty to 35 fractions were pooled (around 300 ml). The sample was dialyzed against an equilibration Q buffer (50 mM NaCl and 2 M Urea in 50 mM Tris buffer) at pH 8.0 for application to the Q-Sepharose FF column (cat. # 17-0510-01; 100 ml resin bed; GE Healthcare Bio-Sciences).

The dialyzed sample was loaded on the Q-Sepharse FF column (GE Healthcare Bio-Science), a strong anion exchanger that had been equilibrated with 10 column volumes of Q buffer. The column was washed with 20 column volumes of wash buffer (150 mM NaCl, 2 M Urea, and 50 mM Tris-HCl at pH 8.0). The bounded proteins were then eluted with 3 column volumes of elution buffer (400 mM NaCl, 2 M Urea, 50 mM Tris-HCl at pH 8.0). The peak fractions were pooled, and dialyzed against PBS. D-(+)-Trehalose (T9531, Sigma) was added to 5% final concentration, and the sample was then frozen at −80°C for storage.

### Assays of protein and endotoxin

The protein content of samples was assayed with the Bradford Method (Quick Start Bradford Protein Assay; BIO-RAD, Hercules, CA). Endotoxin was assayed with the Limulus amoebocyte lysate chromogenic assay (QCL-1000™ Endpoint Chromogenic LAL Assays; Lonza, Walkersville, MD) according to the manufacturer’s instructions. Briefly, 50 μl duplicates of each test sample, 4 standards, and a negative control (apyrogenic LAL water) were transferred to endotoxin free tubes. The samples were incubated at 37°C for an initial period of 10 min, for 10 min after addition of 50 μl LAL lysate, and then for 8 min after gently mixing in 50 μl of substrate. The reaction was terminated with 100 μl of stop reagent. The samples of 200 μl of were transferred to a microtiter plate and within 30 min the absorbance at 405 to 410 nm was recorded.

### Deglycosylation of rhTSG-6

N-Glycosidase A (5 mU; Roche Life Science, Indianapolis, IN) was diluted with 100 mM sodium acetate buffer (pH 5.0) to a concentration of 0.25 mU per 16 μl. rhTSG-6 (1.2 μg) was added, the sample volume was increased to 20 μl with the enzyme diluent, and it was incubated at 37°C for 15, 60, and 180 min. Half of the reaction solution was separated by 10% SDS-polyacrylamide electrophoresis and stained with Coomassie Brilliant Blue solution. The remainder was separated by 10% SDS-polyacrylamide electrophoresis and transferred to nitrocellulose (NC) membrane for staining glycoproteins (Pierce Glycoprotein Staining Kit; cat. # 24562; Thermo Scientific, Rockford, IL). In brief, the NC membrane was immersed in 10 ml 3% acetic acid for 10 min and then transferred to 10 ml of oxidizing solution. After 15 min gentle agitation, the membrane was washed with 10 ml of 3% acetic acid solution for 5 min three times. The membrane was soaked in 10 ml of Glycoprotein Staining reagent for 15 min and then transferred to 10 ml of Reducing Solution and gently washed with 3% acetic acid for 5 min three times. To visualize the stained bands, the membrane was washed with ultrapure water. The stained membrane was stored in 3% acetic acid solution.

### Half-lives of rhTSG-6 in mice

Male C57BL/6 mice 7–8 weeks old (20–22 g) were purchased from The Jackson Laboratory in Bar Harbor, Maine, United States. 50 μg of myeloma cell-extracted rhTSG-6 (R&D systems, 2104-TS) or CHO cell-derived rhTSG-6 were injected into the tail vein. Blood was collected by cardiac puncture at 0, 5 min, 30 min, 1 h, 3 h, 6 h, 12 h, and 24 h after rhTSG-6 injection. To collect blood, the mice were anesthetized by intraperitoneal administration of 100 mg/kg of ketamine and 10 mg/kg of xylazine and blood was recovered in heparin coated capillary blood collection tubes (Terumo), and it was centrifuged at 3,000 × g for 10 min at 4°C. The centrifugation step was repeated twice to minimize platelet contamination and the clear plasma fraction was stored at −80°C. rhTSG-6 protein levels in plasma were determined by ELISA (above). The data were fitted to two compartment models and the half-lives of distribution (t_1/2α_) and elimination (t_1/2β_) were calculated by non-linear least squares regression using GraphPad Prism.

### LPS-induced inflammation model

Mice were randomized to receive intravenously either PBS or rhTSG-6 (25 μg or 50 μg) mixed with 60 μg of LPS from *Escherichia coli* 055:B5 (Sigma, L2880) to induce inflammation. After 6 hours, mice were euthanized by lethal dose of 5% isoflurane in 100% oxygen followed by cervical dislocation. Spleens were collected and frozen in dry ice and stored at −80°C before analysis. RNA was extracted from spleen (RNeasy Mini Kit; cat. # 74106; QIAGEN, Valencia, CA). Approximately 1 μg of total RNA was used to synthesize double-stranded complementary DNA by reverse transcription (Super Script Ⅲ, Life Technologies). The complementary DNA was analyzed by real-time PCR (7900HT Fast Real-Time PCR System; Thermo Fisher Scientific, Carlsbad, CA). For assays for mouse-specific transcripts, mouse-specific primers and probes (Thermo Fisher Scientific) were used: IL-6 (Mm00439653_m1) and IFN-γ (Mm00599890_m1). For relative quantitation of gene expression, mouse-specific GAPDH primer and probes (Mm99999915_g1) were used.

### Statistical analyses

All data analyses were carried out using GraphPad Prism software (GraphPad Software, Inc., La Jolla, CA). Continuous variables were tested for normal distribution by using the Kolmogorov-Smirnov (KS) test. For comparison of two groups of non-normally distributed data was performed using Mann-Whitney U test. Comparisons of parameters among the three or more groups were made by one-way ANOVA followed by the Tukey or Newman-Keuls multiple comparison post hoc test for normally distributed data. A value of *p* < 0.05 was considered statistically significant.

## Results

### Preparation of Stable Lines of CHO Cells Expressing rhTSG-6

**Fig 1:**
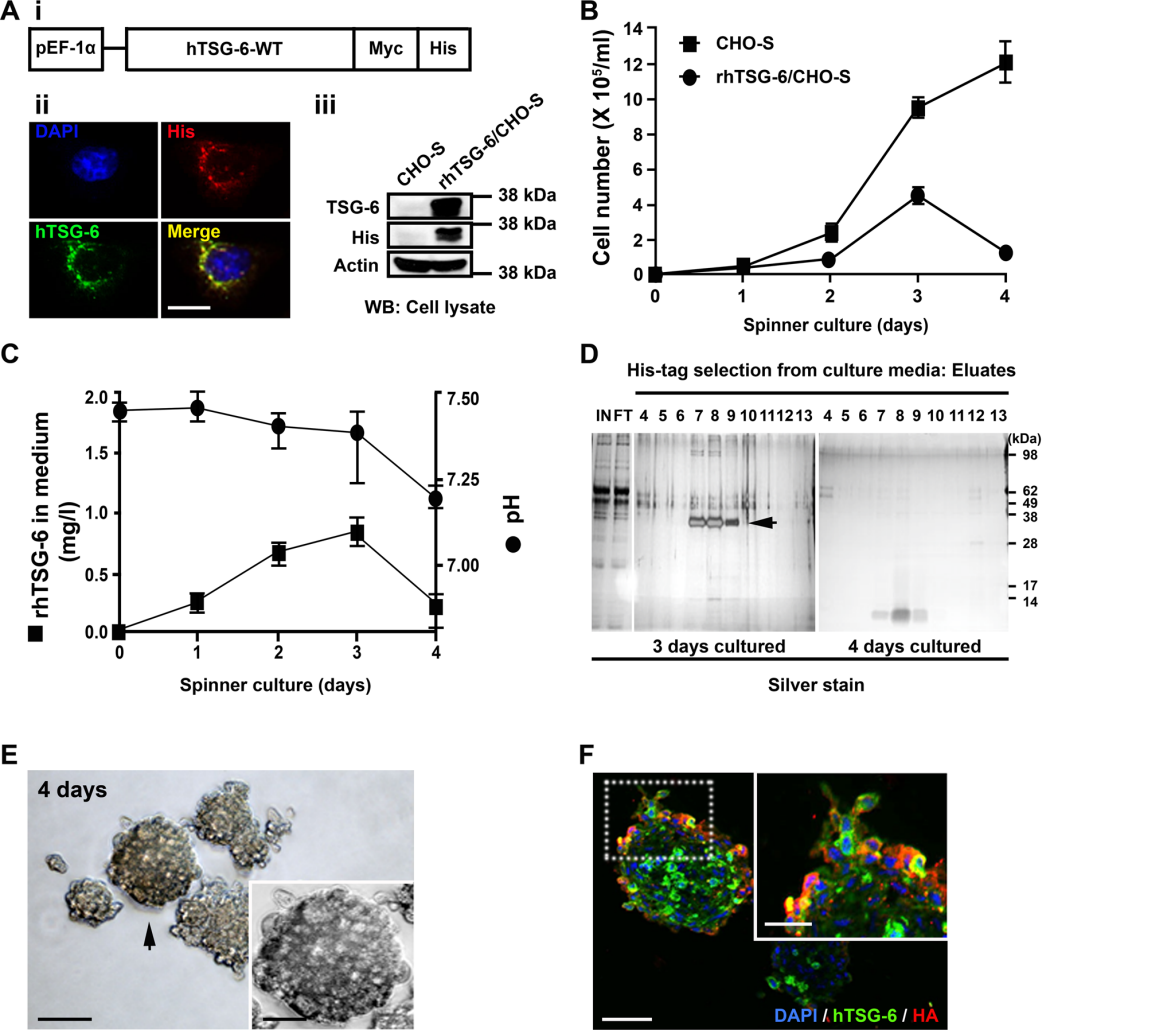
CHO cell line stably transfected to synthesize rhTSG-6 forms aggregates resulting in a decrease of protein production. Cells were incubated in CD CHO basal media and in spinner culture. (A i) Diagram of expression plasmid of TSG-6 under control of human elongation factor promoter 1α and insertion of sequences for Myc and His tags at the C-terminus. (A ii) Expression of rhTSG-6 (antibody clone A38.1.20) and the His tag by immunocytochemistry. Scale bar = 10 μm. (A iii) Expression by Western blotting. (B) Number of control CHO cells (CHO-S) and rhTSG-6 synthesizing cells (rhTSG-6/CHO-S). Data are presented as mean ± S.D. (C) rhTSG-6 content and pH of medium from cultures of rhTSG-6/CHO-S cells. Data are presented as mean ± S.D. (D) Western blots with antibodies to the His-tag of medium from cultures of rhTSG-6/CHO-S cells. Monomeric rhTSG-6 was detected after day 3 but not after day 4, apparently because of aggregation. Abbreviations: IN, input sample; FT, flow through. (E) Aggregates of rhTSG-6/CHO-S cells after 4 days of culture. Scale bars = 500 μm and 250 μm (insert). (F) Immunolabeling with rhTSG-6 antibody (clone A38.1.20), HABP (biotin-conjugated HA binding proteins), and DAPI staining of aggregates. Scale bars = 100 μm and 50 μm (insert).

A DNA construct containing sequences for human wild type TSG-6 [5, 36] was prepared from RNA extracted from human MSCs incubated with TNF-α to increase expression of TSG-6 [32]. We elected to clone the sequences into a plasmid with a promoter of elongation factor EF-1α to avoid the silencing occasionally encountered with other promoters (Fig 1A, i). The plasmid also incorporated Myc-tag and His-tag of sequences at the C-terminus to facilitate detection and purification of the proteins. After the cDNAs were sub-cloned into the plasmid (Fig 1A, i), the plasmid was amplified in *E*. *coli*, and the insert was sequenced to verify its structure (not shown). An expression plasmid was then used to prepare stable transfectants of CHO cells using a lipofectamine protocol. Expression of rhTSG-6 was confirmed by Western blot and immunostaining for rhTSG-6 and the C-terminal His-tag after transient transfection to CHO-S cells (Fig 1A, ii, iii). For isolation of clones, the cells were cultured on a methylcellulose medium containing 800 μg/ml of Zeocin that makes it possible to isolate clones in 2 to 3 weeks [41, 42]. To identify clones that secreted the recombinant protein, medium from the clones was assayed for expression by Western blotting and the results confirmed by immunocytochemistry of cultures of the clones (S1 Fig). The transfected cells were similar to controls in morphology and the rhTSG-6 was present in the cytoplasm in a punctate distribution consistent with its being processed for secretion (Fig 1A.ii and S1D Fig).

The most highly expressing clones synthesized rhTSG-6 at a rate that was about 100-fold greater than the rate previously obtained with human MSCs that were shown to secreted relatively large amount of rhTSG-6 after they were activated by incubation with TNF-α [32], i.e. about 500 ftmoles of rhTSG-6 /10^6^ CHO cells/ 24 h versus 5 ftmoles by human MSCs incubated with TNF-α.

### Optimization of Conditions for Production of TSG-6 in a Spinner Culture

For production of the recombinant protein, we first incubated the expanded CHO clones in a spinner culture flask with a commercially available chemically-defined and protein-free medium for CHO cells (CD-CHO Medium; Invitrogen). As indicated in Fig 1B, the transfected cells expanded but at a slower rate than untransfected CHO-S cells. Surprisingly, however, the yield of the transfected cells sharply decreased between day 3 and day 4. Also, there was a sharp decrease in the amount of rhTSG-6 recovered from the medium (Fig 1C) with monomeric protein detected in the medium after 3 days but essentially none after 4 days, apparently because of aggregation (Fig 1D). Similar results were obtained in 5 similar experiments (not shown). Microscopy of the cultures demonstrated that between days 3 and 4, the CHO formed large clusters of cells (Fig 1E). As expected, immunocytochemistry demonstrated that the aggregates contained both rhTSG-6 and hyaluronan binding protein (Fig 1F). The decrease in yield of both cells and rhTSG-6 after day 3 was therefore explained by the well-documented tendency of the protein to bind hyaluronan [8, 43] and the fact that the CHO cells, like many cells in culture, are surrounded by a brush border of hyaluronan [44]. The affinity of TSG-6 increases at acidic pHs with a peak at about pH 6 [45–47]. Therefore, the tendency of the rhTSG-6 to aggregate CHO cells in the spinner cultures was enhanced by the decrease in pH (Fig 1C) that probably reflected decreased gaseous exchange and an accumulation of CO_2_ in the medium [48].

### Conditions for Decreasing Aggregation of CHO Cells

**Fig 2:**
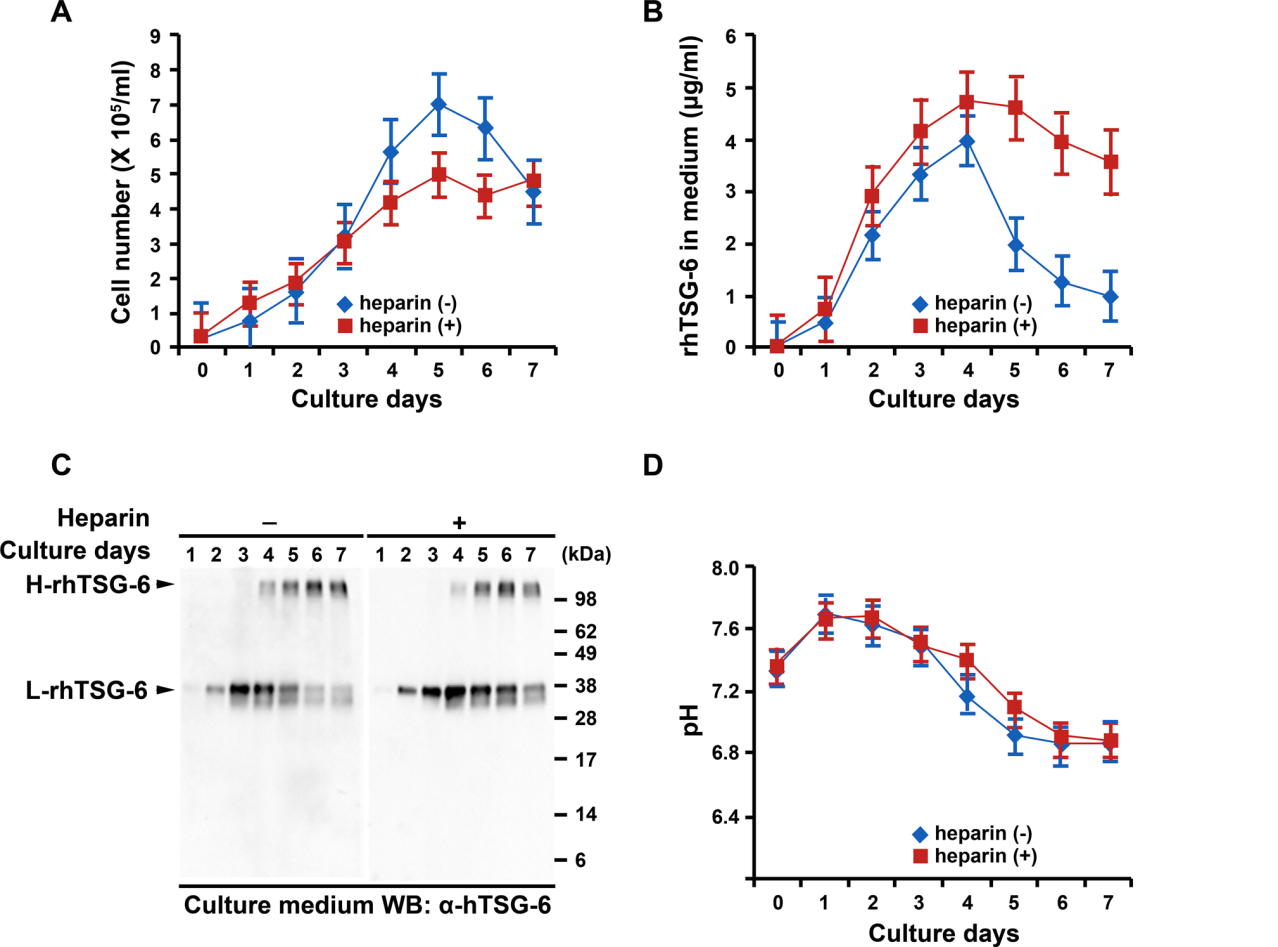
Addition of heparin to medium improved yield of rhTSG-6 in spinner cultures. (A) Effect on number of rhTSG-6/CHO-S cells. (B) Effect on yield of rhTSG-6. (C) Effect on yields of protein aggregates/complexes (H-rhTSG-6) and monomeric rhTSG-6 (L-rhTSG-6). Western blots with antibodies to hTSG-6 on medium from day 4 cultures of rhTSG-6/CHO-S cells. (E) Effects on pH of medium. Values are mean ± S.D. of 3 replicates of each sample in (A), (B), and (D).

To reduce the tendency of the rhTSG-6 to cause aggregation of the CHO cells, we instituted two measures. One was addition to the medium of an inhibitor of hyaluronan synthesis, 4-methylumbelliferone [38], at a concentration of 50 μM (see S3 Fig). The second measure was to add heparin (Table 1) to the medium to compete with the binding of rhTSG-6 to hyaluronan [10, 49]. The addition of heparin to the spinner cultures improved the yield of cells (Fig 2A) and production of rhTSG-6 (Fig 2B) [39]. It also increased slightly the amount of protein that was recovered in a monomeric form and correspondingly decreased the aggregated form (Fig 2C). But addition of heparin to spinner culture did not prevent the decrease in pH (Fig 2D), an observation suggesting that better control of pH would be helpful.

We also instituted several additional conditions to improve the yield. We supplemented the medium with hypoxanthine/thymidine (Table 1) as suggested by Chen et al., [50] to increase the yield of cells and recombinant proteins (not shown). We further supplemented the medium with a high level of glucose (Table 1) to improve cell yields (S2A Fig). Separate additions of a lipid concentrate or a surfactant polymer (Pluronic F68) also improved cell yield but, surprisingly, a combination of the two inhibited the system (S2C Fig). Therefore, these supplements were omitted. Supplementation with non-essential amino acids (Table 1) protected against the decrease in cell yield between days 3 and 4 (compare S2A and S2B Fig). The results of the experiments provided a medium that was defined here as optimized chemically-defined and protein-free medium (OCDPF) (Table 1).

### Scalable Production in a Bioreactor

**Fig 3:**
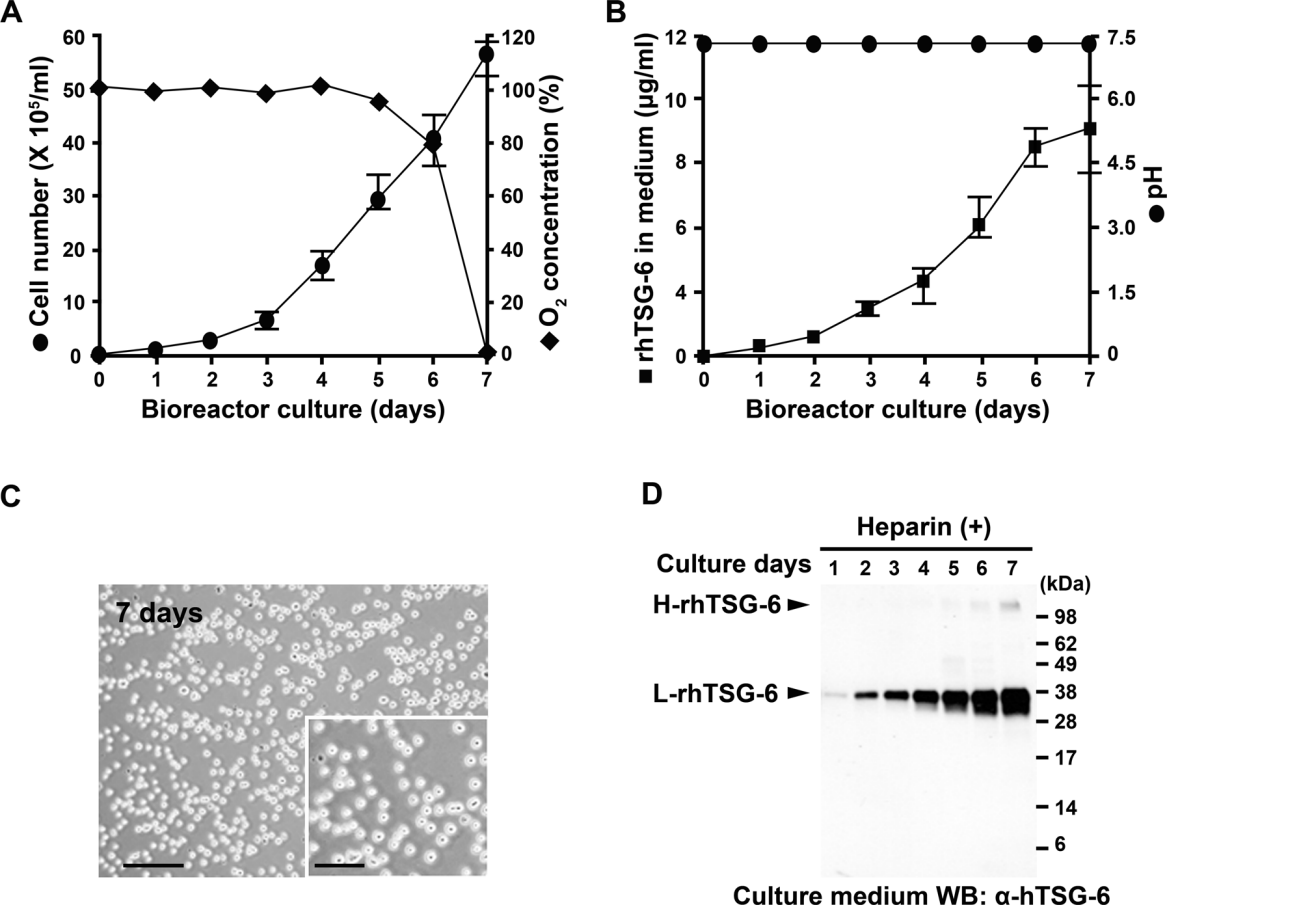
Synthesis of rhTSG-6 by culture of rhTSG-6/CHO-S cells in OCDPF medium and in a bioreactor that controlled pH. (A) Expansion of cells and oxygen saturation. (B) Yield of rhTSG-6 and pH of medium. (C) Photomicrographs demonstrating that rhTSG-6/CHO-S cells are largely single-cell suspensions. Scale bars = 100 μm and 50 μm (insert) (D) Yield of monomeric rhTSG-6. Western blot of medium with antibodies to hTSG-6. Values in (A) and (B) are means of 3 replicates.

In addition to the above measures, we elected to replace the spinner culture system with a bioreactor that allowed control of pH and oxygen. Under the optimized conditions in the bioreactor, we were able to increase the cell number almost 10-fold (from about 7 × 10^5^ per ml in Fig 2A to about 60 × 10^5^ per ml in Fig 3A). However, the bioreactor did not provide complete control of the incubation system, since the oxygen concentration fell drastically on day 7 (Fig 3A), apparently because of the frequently encountered problem of insufficient gaseous exchange as the cell concentration increased [48]. The bioreactor did control pH (Fig 3B). Most importantly, the yield of TSG-6 in medium increased with time in culture for 6 or 7 days (Fig 3B). In addition, the CHO cells did not aggregate (Fig 3C) and most of the TSG-6 in the medium was recovered in a monomeric form (Fig 3D).

### Purification of the rhTSG-6

**Fig 4:**
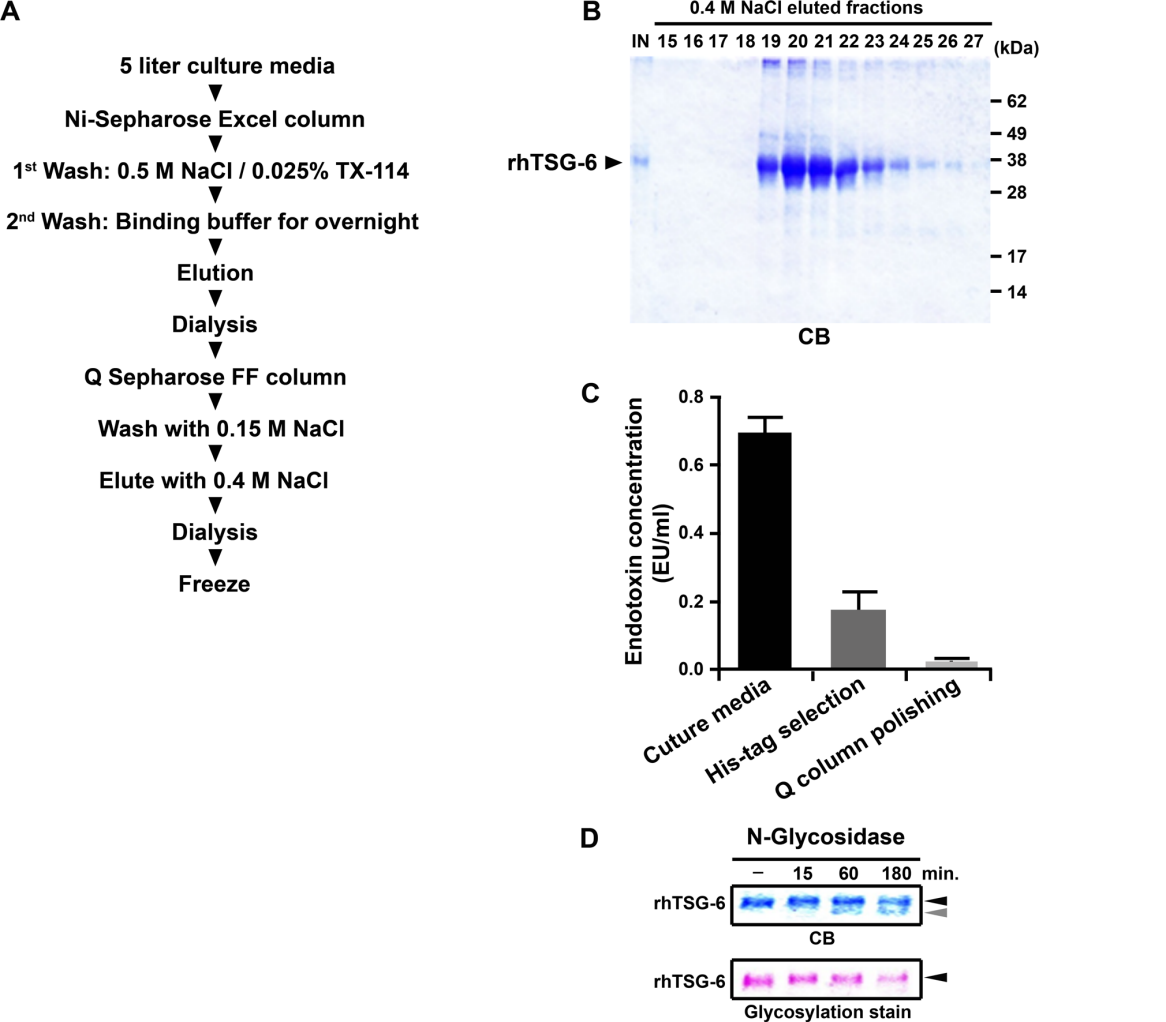
Purification of rhTSG-6 from 5 or 6 liters cultured media of the bioreactor. (A) Schematic for the purification steps. (B) Assay by SDS-PAGE of rhTSG-6 eluted from the Q-Sepharose FF column. Symbols: IN, input sample; CB, Coomassie Blue staining of gel. (C) Endotoxin content of conditioned culture medium, eluate from the His-tag column and Q-Sepharose FF column. Values are mean ± S.D. of 3 replicates. (D) Deglycosylation of the purified rhTSG-6. Arrows indicate deglycosylated forms rhTSG-6.

In initial experiments to purify the rhTSG-6, we observed that the protein tended to self-aggregate in an apparently irreversible manner at 4°C. Therefore, we stored the culture medium at -20°C, but after thawing, purified the protein at room temperature. As a first step in purification, the culture medium was chromatographed on a Ni-chelate column (Fig 4A) to take advantage of the His-tag engineered into the C-terminus of the protein (Fig 1A). After binding of the protein, the column was washed with Triton X-114, a step shown to remove endotoxin [51]. The rhTSG-6 was then eluted and the protein chromatographed on an anion exchange column. The isolated protein was homogeneous as assayed by stained electrophoretic gels and primarily in a monomeric form (Fig 4B). The initial levels of endotoxin of about 0.7 EU/ml were below recommendation limits for preclinical research [52] and they were lower in the isolated protein (Fig 4C). A concentration of 0.025% Triton X-114 was used for the final protocol (S4 Fig) to provide purified rhTSG-6 with 0.375~0.625 EU/mg of rhTSG-6 without sacrificing the recovery of rhTSG-6. The FDA threshold for the pyrogenic human dose of 5 EU per kg for preclinical research [52] and therefore would permit a dose of up to 7 mg/kg of the purified rhTSG-6.

In addition, digestion with N-glycosidase indicated that it was glycosylated (Fig 4D). As expected, the apparent molecular weight and the specific staining of the protein decreased with deglycosylation.

The purification of the rhTSG-6 is summarized in Table 2. Of interest was that the yields of rhTSG-6 in the recovered CHO medium were 57.0 mg ± 9.16 S.D. in 5 or 6 liters of medium and that rhTSG-6 accounted for 18.0% ± 3.76 S.D. of the total protein in the medium. A 4- to 5-fold purification was obtained with chromatography on the Ni-chelate column with a recovery of 55.0% ± 12.3 S.D. The polishing step on the anion exchange column provided a higher yield and the over-all yield from the culture medium was 40.6% and 47.2% in 2 purification runs. After elution from the anion exchange column, the protein was dialyzed against PBS, trehalose was added to a final concentration of 5%, and it was stored at -20°C. Samples stored at -20°C for up to 3 months and then thawed were monomeric by gel electrophoresis (as in Fig 4B) and were active in suppressing inflammation in vivo in the LPS mouse model (see below).

**Table 2:** Synthesis and Purification of rhTSG-6 in Bioreactor.

Sample	Volume (ml)	Protein (mg)	rhTSG-6 (mg)	Over-all recovery of rhTSG-6	Ratio of rhTSG-6/ Proteins × 100
^a^OCDPF culture medium	5,000 or 6,000	321.7±36.4^b^ (n = 6)	57.0±9.16 (n = 6)	-	18.0±3.76% (n = 6)
Ni-Sepharose Excel eluate	231.3±5.65 (n = 6)	35.4±3.53 (n = 6)	29.7±2.82 (n = 6)	55.0±12.3% (n = 6)	83.9±5.2% (n = 6)
Q Sepharose FF eluate	56, 56	21.5, 33.5	20.6, 33.4	40.6, 47.2%	95.8 to 99.7% (n = 2)

^a^ Optimized chemically-defined proteins free medium with supplements indicated in Table 1.

^b^ Standard deviation.

### Metabolic Half-Life of the rhTSG-6

**Fig 5:**
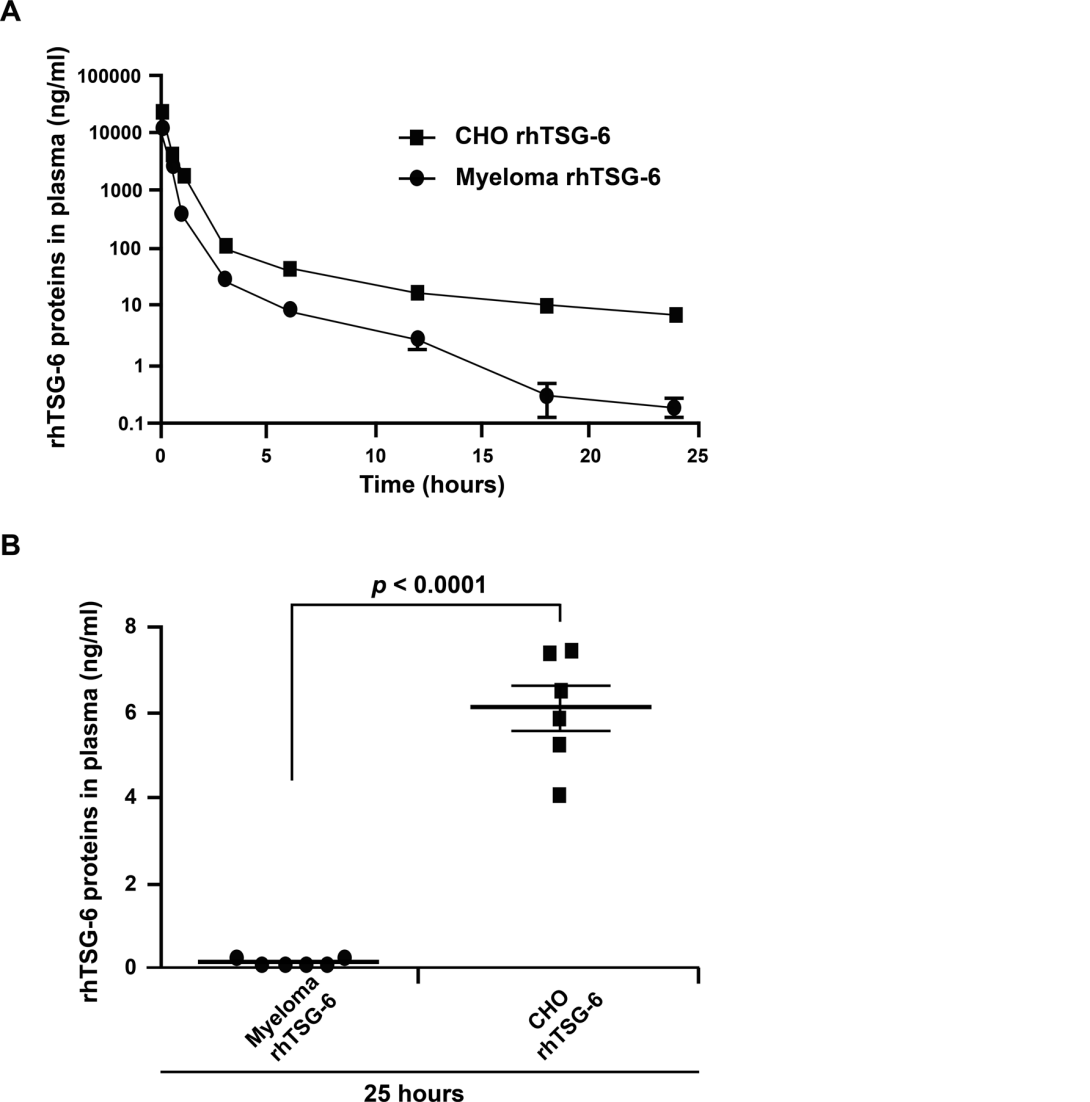
*In vivo* half-life of rhTSG-6 proteins in plasma of mice. (A) rhTSG-6 (50 μg) was injected through the tail vein and the blood was collected at times indicated. After isolation of the plasma, levels of rhTSG-6 proteins were determined by ELISA. Values are mean and SEM from 3 mice. Distribution (t_1/2α_) and elimination (t_1/2β_) were calculated by non-linear least squares regression using GraphPad Prism program. Myeloma-derived rhTSG-6 (R&D Systems): t_1/2α_ = 0.15 h, t_1/2β_ = 0.20 h, CHO cell-derived rhTSG-6: t_1/2α_ = 0.08 h, t_1/2β_ = 0.47 h. The results showed a tendency for the CHO cell-derived rhTSG-6 to have a longer half-life. (B) The difference plasma levels at 24 h after infusion of 50 μg rhTSG-6 of the two proteins. Comparison of the two groups was made by Student’s t test with Welch’s correction. Data are presented as mean ± S.E.M. from 6 mice.

One measure of the biological activity of a secreted glycoprotein like TSG-6 is its metabolic half-life in vivo, since misfolded and unglycosylated proteins tend to be cleared rapidly [53]. Therefore, we injected 50 μg of the protein through the tail veins of C57BL/6 mice and assayed the metabolic half-life in plasma. As indicated in Fig 5, the t_1/2_ for the initial distribution phase of the rhTSG-6 synthesized here was 0.08 h and the elimination phase was 0.47 h. The corresponding values for a commercially available rhTSG-6 (R&D Systems) were 0.15 h and 0.20 h. The results showed a tendency of the CHO cell-derived TSG-6 to have a longer half-life. The tendency was confirmed in a further experiment in which a larger number of mice were compared 25 h after infusion of the two proteins (Fig 5B). The differences are probably explained by the fact that although both proteins were monomeric as assayed by SDS-PAGE, the rhTSG-6 from the CHO cells is a secreted glycosylated form of the protein whereas the commercial rhTSG-6 was synthesized in mouse myeloma cells and was purified from cell lysates.

### Suppression of Inflammation in Vivo

**Fig 6:**
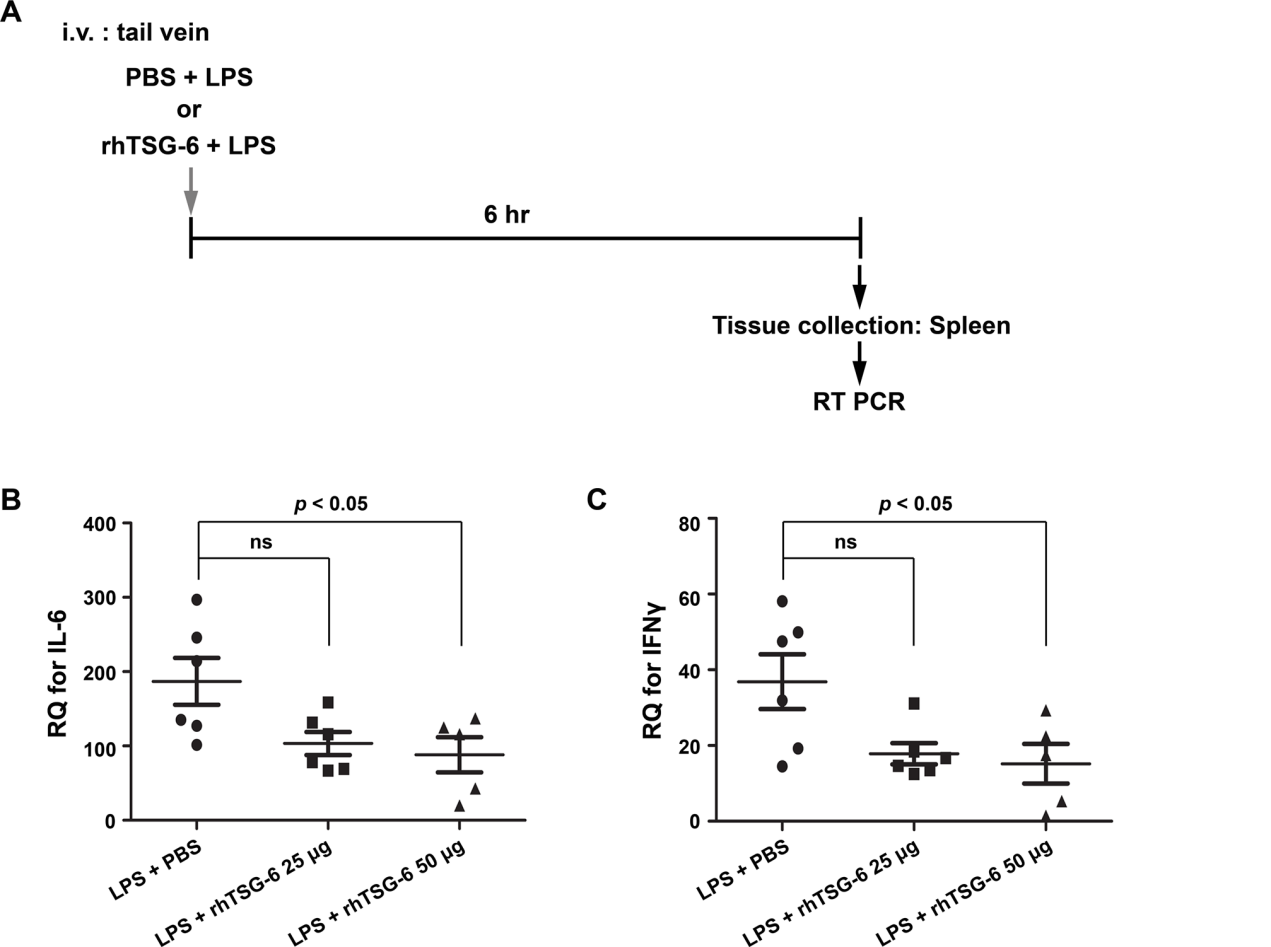
Purified rhTSG-6 suppressed LPS-induced inflammation in mice. (A) Schematic for the experiment. (B, C) rhTSG-6 suppressed LPS-induced levels of mRNA for IL-6 and IFNγ in spleen. Statistical calculation was performed by a one-way ANOVA followed by the Tukey multiple comparison post hoc test. Data are presented as mean ± S.E.M. Symbol: RQ, relative quantity.

TSG-6 was shown to suppress inflammation in a series of different animal models [see 25]. Most of the models require some technical expertise. Therefore, we elected to test the rhTSG-6 in a simple model in which inflammation is produced in mice by intravenous injection of the bacterial extract LPS and RT-PCR assays are used to detect the increase in expression of pro-inflammatory cytokines in spleen. As indicated in Fig 6, there was a large variance in the assay. Also, the maximum amount of rhTSG-6 testable was limited by the solubility limit of the protein (about 0.5 μg per μl). However, the data indicated that intravenous administration of 50 μg of rhTSG-6 from CHO cells suppressed the LPS-induced mRNA levels for both IL-6 and IFNγ in spleen (*p*<0.05). The results suggested but did not conclusively establish the effect was dose dependent since 25 μg of rhTSG-6 did not produce a significant change in the Tukey post hoc test but did in the Newman-Keuls multiple comparison post hoc test. The similar results were obtained with a control of a commercially available rhTSG-6 in the same assay (S6 Fig).

## Discussion

Synthesis of rhTSG-6 has been an unmet challenge since the protein was first discovered over 20 years ago [4]. Limited amounts were produced in several systems, but scalable production was not achieved. The results presented here demonstrate that cellular synthesis of rhTSG-6 is limited by well-known characteristics of the protein. It belongs to a family of hyaluronan binding proteins, termed hyadherins, that extensively interact with proteins in the extracellular matrix [46]. As part of its interaction with matrix components, TSG-6 forms cross-links with hyaluronan either by binding directly to the linear high molecular weight glycosoaminoglycan or by serving as a cofactor and catalyst for the covalent transfer of heavy chains from inter-α-inhibitor to hyaluronan [43]. As demonstrated here, these properties limit production of the recombinant protein by CHO cells because the rhTSG-6 bound to and probably cross-linked the hyaluronan that forms a brush found on most cultured cells [44]. As a result, the CHO cells formed large aggregates which limited both the propagation of the cells and the amount of rhTSG-6 recovered from the medium. In addition, the tendency of the protein to self-aggregate presented a serious technical challenge. The aggregates may have retained biological activity but they were too difficult to isolate and assay. Also, the aggregates were not dissociated by standard conditions for SDS-PAGE. Instead, they required more stringent conditions for dissociation (see S5 Fig), an observation occasionally made with other protein aggregates (see ref. 41).

The protocol developed here largely surmounts these problems. The dramatic tendency of the CHO cells to be aggregated by the newly-synthesized rhTSG-6 was reduced by use of an inhibitor of hyaluronan synthesis and addition of heparin as competitor for the binding of TSG-6 to hyaluronan [10]. Optimization of a standard medium for culture of CHO cells increased production. To prevent aggregation, procedures to concentrate the medium were avoided and the purification steps were performed at room temperature. The use of the His-tag to isolate the protein from the medium was more feasible than using antibodies because the available antibodies to TSG-6 are not highly specific and purification with antibodies is usually less cost-effective. Since rhTSG-6 accounted for 18.0% ± 3.76 S.D. of the total protein in the medium, chromatographic purification without resort to the His-tag may be effective.

The conditions developed here to produce rhTSG-6 may be useful to produce recombinant forms of other extracellular proteins that tend to aggregate with themselves or the cells that secrete them, proteins such as pentraxins, fibronectin, and thrombospondins.

The yields of rhTSG-6 obtained here in a laboratory scale bioreactor of 5 to 6 liter should be adequate to allow, for the first time, structure studies on TSG-6 similar to the structural studies performed with the N-terminal half of the protein that contained the hyaluronan binding domain and that was synthesized in bacteria [36, 49]. It should also be adequate for extensive studies in mouse and rat models for human diseases. The amounts may also be adequate for local administration in larger animals and human subjects for conditions such as corneal defects and joint injuries or diseases. Still larger amounts will probably be required for systemic administration in large animals and human subjects, but the present protocol was designed to be scalable for production in large bioreactors and purification with chromatographic systems that can be readily enlarged. Therefore, it should be feasible to overcome the problems of scale that limited earlier attempts to use the protein for clinical therapies. Initial experiments in mouse models suggested that the protein would be useful to treat arthritis [26–29]. More recent observations raise the possibility that it may have wide applications in suppressing excessive inflammation in a large number of diseases [25].

## Conclusions

The protocol for preparing recombinant hTSG-6 from CHO overcomes the marked tendency of the protein to aggregate with itself and the cells that synthesize it. The protocol is scalable and therefore makes it possible for the first time to carry out research on the structure and function of the protein that was not previously possible. Also, it will probably make it possible to explore further its important therapeutic potentials.

## Supporting Information

S1 Fig Rapid generation of stable clones of rhTSG-6/CHO using α- methylcellulose-based protocol. (A) Schematic diagram for the generation of the clones. (B) Phase contrast photographs of transfected clones of CHO cells. The cloned CHO cells formed spheres that were up to 500 μm in diameter. (C) Western blots with antibodies to rhTSG-6 (arrows) in medium from stable clones. (D) Immunocytochemistry of an isolated clone labeled with antibodies to rhTSG-6 (α-hTSG-6). Scale bars = 20 μm and 10 μm (insert).
(TIF)

S2 Fig Optimal conditions for culture of stably transfected CHO cells in a chemically defined and protein-free medium. The cells were cultured in 500 ml of medium in spinner bottle cultures. (A) Effects of adding glucose to a concentration to 11 mM. All subsequent trials were with 11 mM glucose. (B) Effect of adding a lipid concentrate (cat. # 11905–031; Invitrogen) and a surfactant (Pluronic F-68; Invitrogen) either separately or together. (C) Effects of adding non-essential amino acids (cat. # 11140–050; Invitrogen). (D) Effect of culture with the optimized chemically-defined and protein-free medium (OCDPF medium) that was developed on the basis of the trial experiments. Values are means of 3 replicates. Similar results were obtained in 3 experiments.
(TIF)

S3 Fig Effects of 4-methylumbelliferone on growth of stable clones of CHO cells. (A) Effects on cell yields in cultures in adherent plates in OCPDF medium. (B) Effects on cell yields in cultures in spinner bottles in OCDPF medium. A concentration of 50 μM was used for OCDPF medium. The data are means and SD of 3 replicates. The *p* values indicated are for data from Day 4 evaluated by 1-way ANOVA.
(TIF)

S4 Fig Optimal concentration of Triton X-114 to remove endotoxin by washing nickel-chelate column. (A) Recovery of rhTSG-6 as a function of Triton X-114 concentration. (B) Endotoxin in the eluted fractions as a function of Triton X-114 concentration.
(TIF)

S5 Fig Dissociation of rhTSG-6 aggregates with NaOH. (A) Schematic of the experiment. (B) The aggregates of rhTSG-6 (H-rhTSG-6) dissociated into monomers (L-rhTSG-6) with 50 mM and 100 mM NaOH.
(TIF)

S6 Fig Comparison of rhTSG-6 synthesized with present protocol and a commercially available rhTSG-6 (R&D Systems). LPS (30 μg) was injected into a tail vein of BALB/C followed immediately by injection of rhTSG-6. Mice were killed 6 h later, spleens harvested and spleens assayed by RT-PCR.
(TIF)

S1 Table Purification process of rhTSG-6 proteins from 5 liters of CHO cell cultured medium. (DOCX)
