# Supplementary figures and images for: Scalable Production of a Multifunctional Protein (TSG-6) That Aggregates with Itself and the CHO Cells That Synthesize It
Source: PLoS One. 2016 Jan 21;11(1):e0147553. doi: 10.1371/journal.pone.0147553 (PMC4721919; doi:10.1371/journal.pone.0147553)

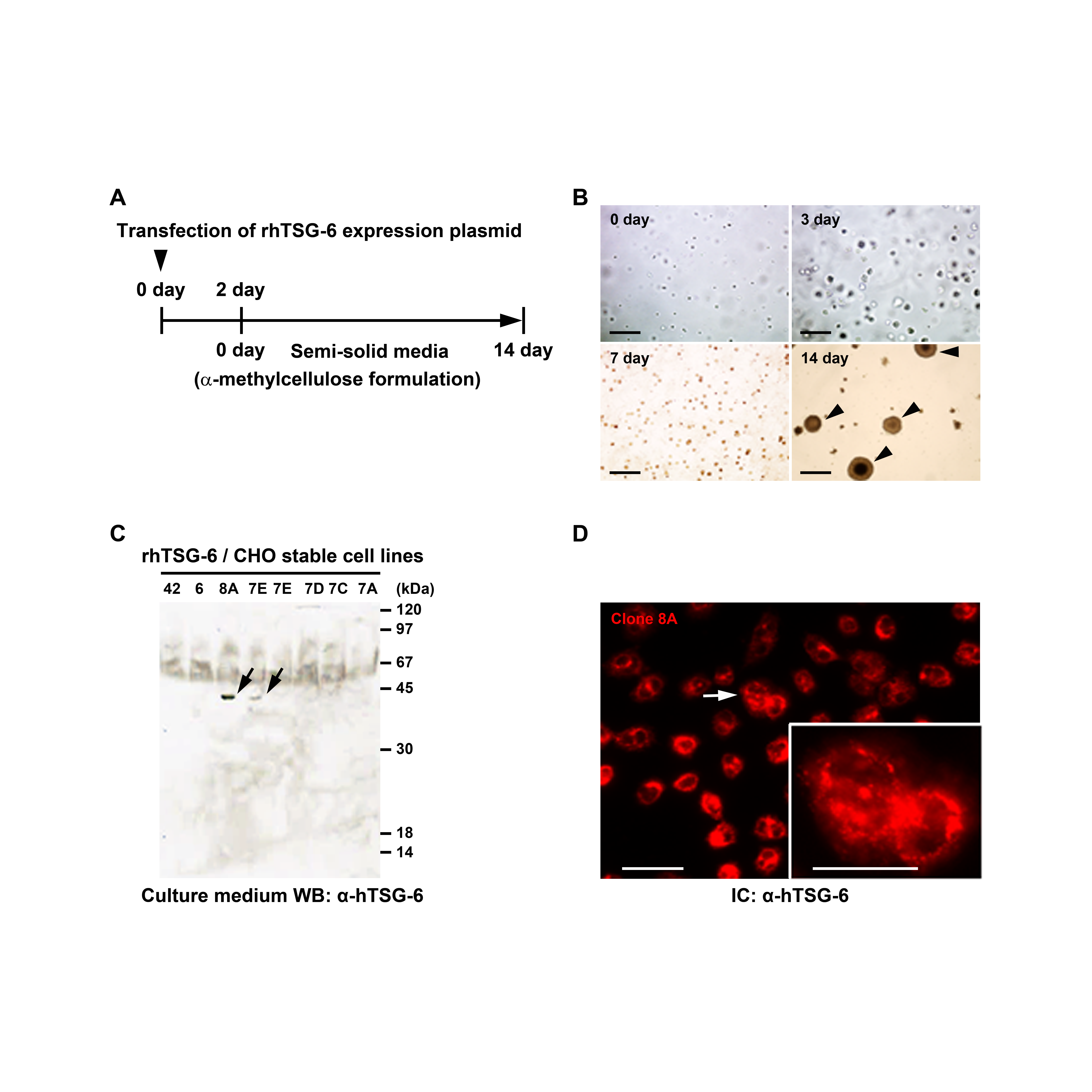

Supplement: S1 Fig — (A) Schematic diagram for the generation of the clones. (B) Phase contrast photographs of transfected clones of CHO cells. The cloned CHO cells formed spheres that were up to 500 μm in diameter. (C) Western blots with antibodies to rhTSG-6 (arrows) in medium from stable clones. (D) Immunocytochemistry of an isolated clone labeled with antibodies to rhTSG-6 (α-hTSG-6). Scale bars = 20 μm and 10 μm (insert). (TIF) [file pone.0147553.s001.tif]

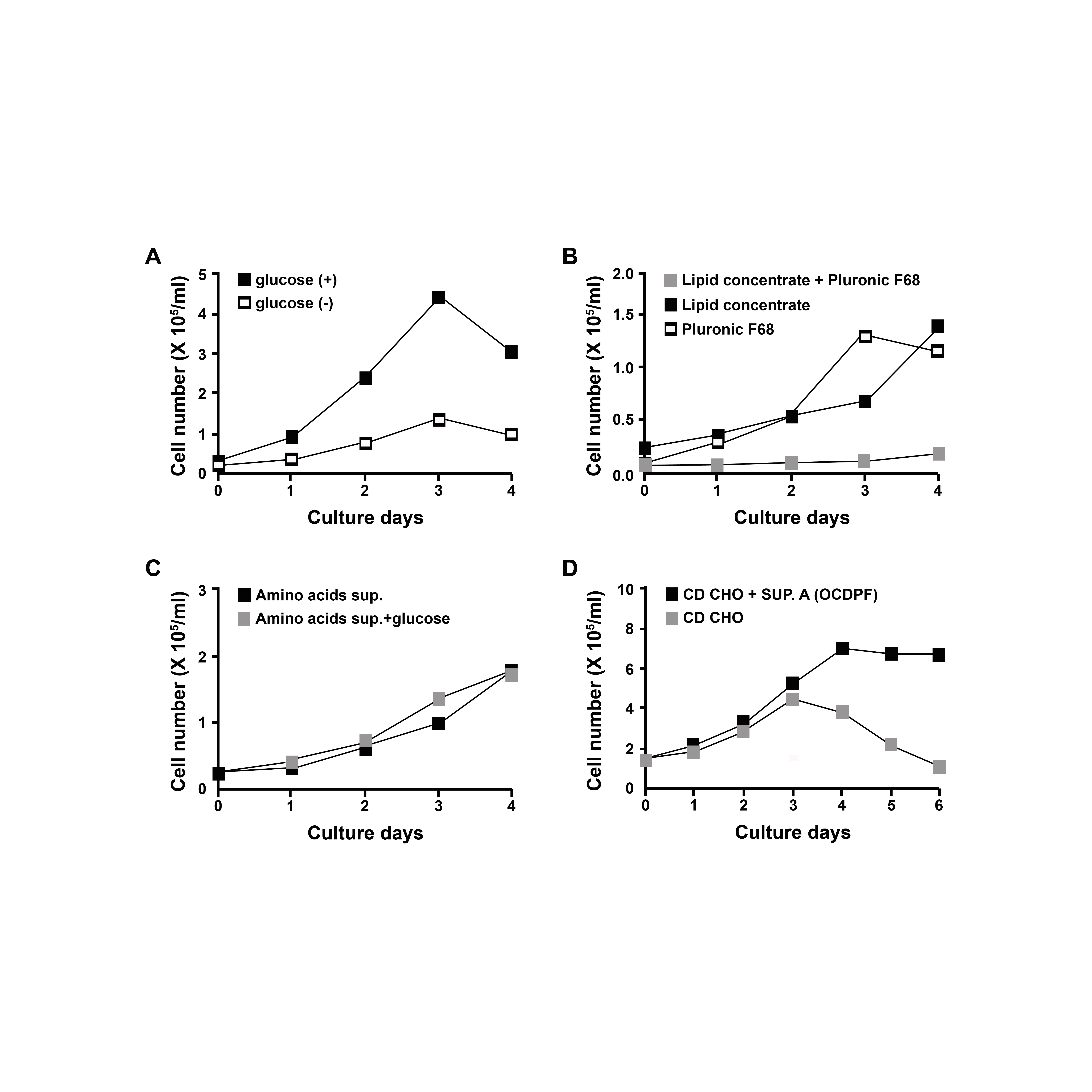

Supplement: S2 Fig — The cells were cultured in 500 ml of medium in spinner bottle cultures. (A) Effects of adding glucose to a concentration to 11 mM. All subsequent trials were with 11 mM glucose. (B) Effect of adding a lipid concentrate (cat. # 11905–031; Invitrogen) and a surfactant (Pluronic F-68; Invitrogen) either separately or together. (C) Effects of adding non-essential amino acids (cat. # 11140–050; Invitrogen). (D) Effect of culture with the optimized chemically-defined and protein-free medium (OCDPF medium) that was developed on the basis of the trial experiments. Values are means of 3 replicates. Similar results were obtained in 3 experiments. (TIF) [file pone.0147553.s002.tif]

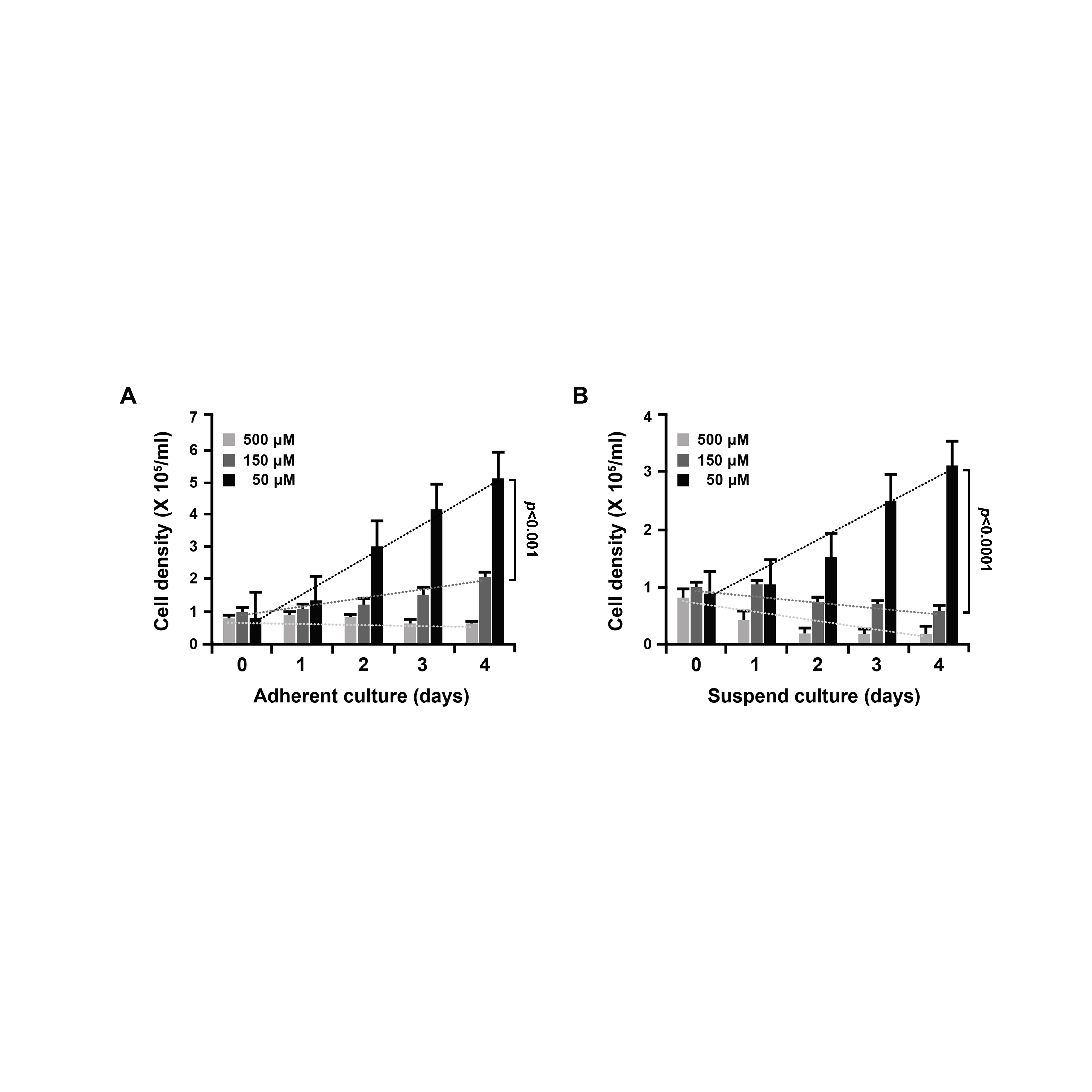

Supplement: S3 Fig — (A) Effects on cell yields in cultures in adherent plates in OCPDF medium. (B) Effects on cell yields in cultures in spinner bottles in OCDPF medium. A concentration of 50 μM was used for OCDPF medium. The data are means and SD of 3 replicates. The p values indicated are for data from Day 4 evaluated by 1-way ANOVA. (TIF) [file pone.0147553.s003.tif]

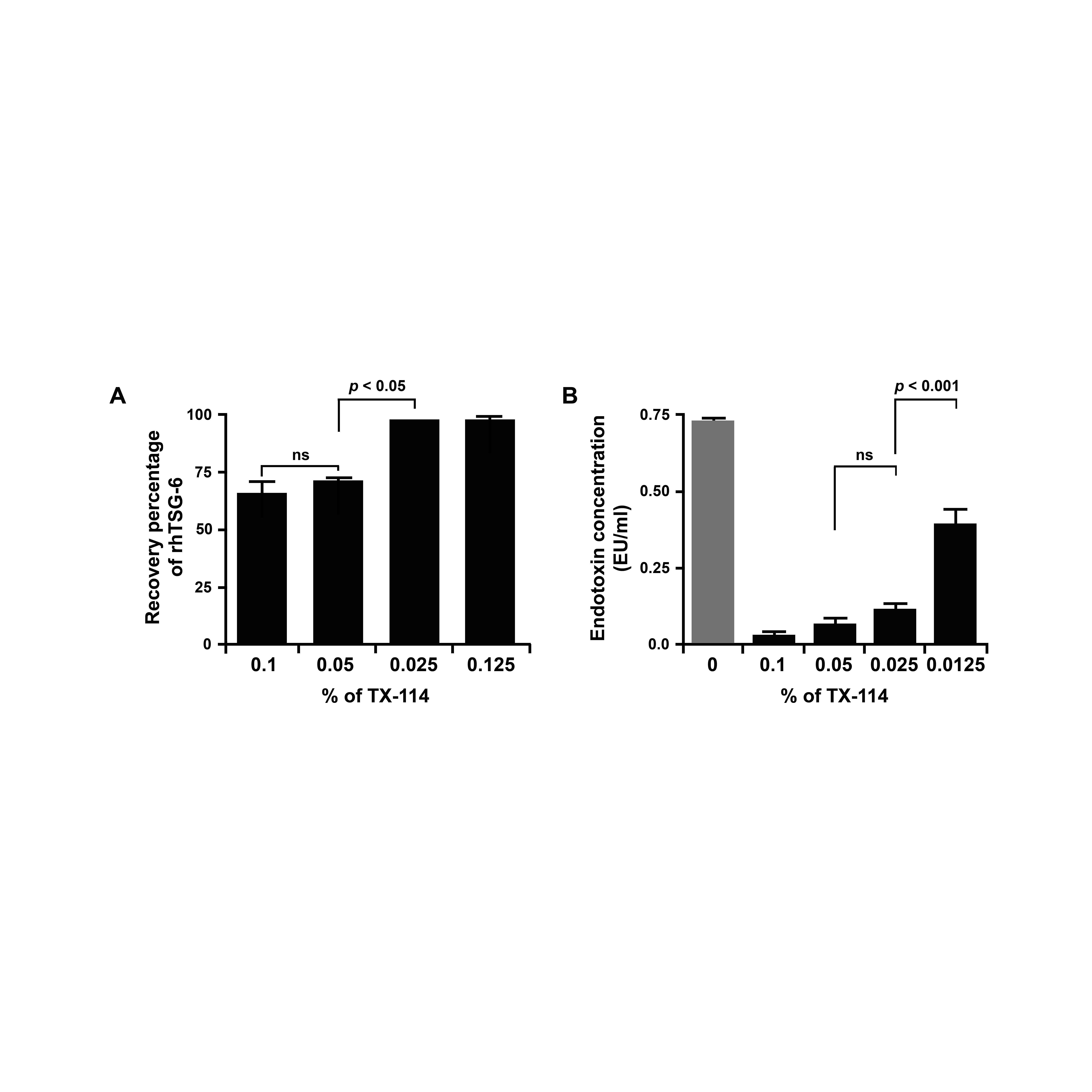

Supplement: S4 Fig — (A) Recovery of rhTSG-6 as a function of Triton X-114 concentration. (B) Endotoxin in the eluted fractions as a function of Triton X-114 concentration. (TIF) [file pone.0147553.s004.tif]

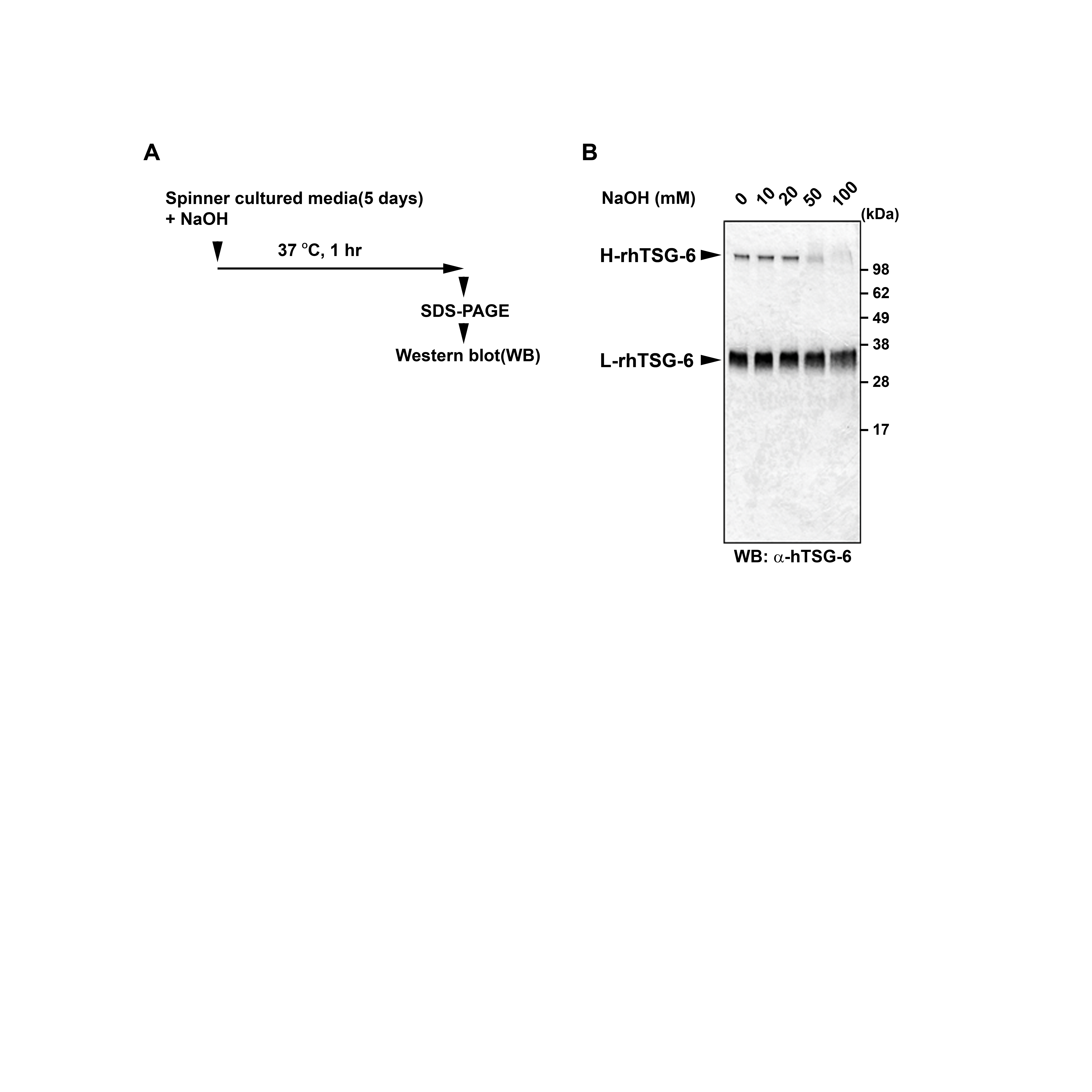

Supplement: S5 Fig — (A) Schematic of the experiment. (B) The aggregates of rhTSG-6 (H-rhTSG-6) dissociated into monomers (L-rhTSG-6) with 50 mM and 100 mM NaOH. (TIF) [file pone.0147553.s005.tif]

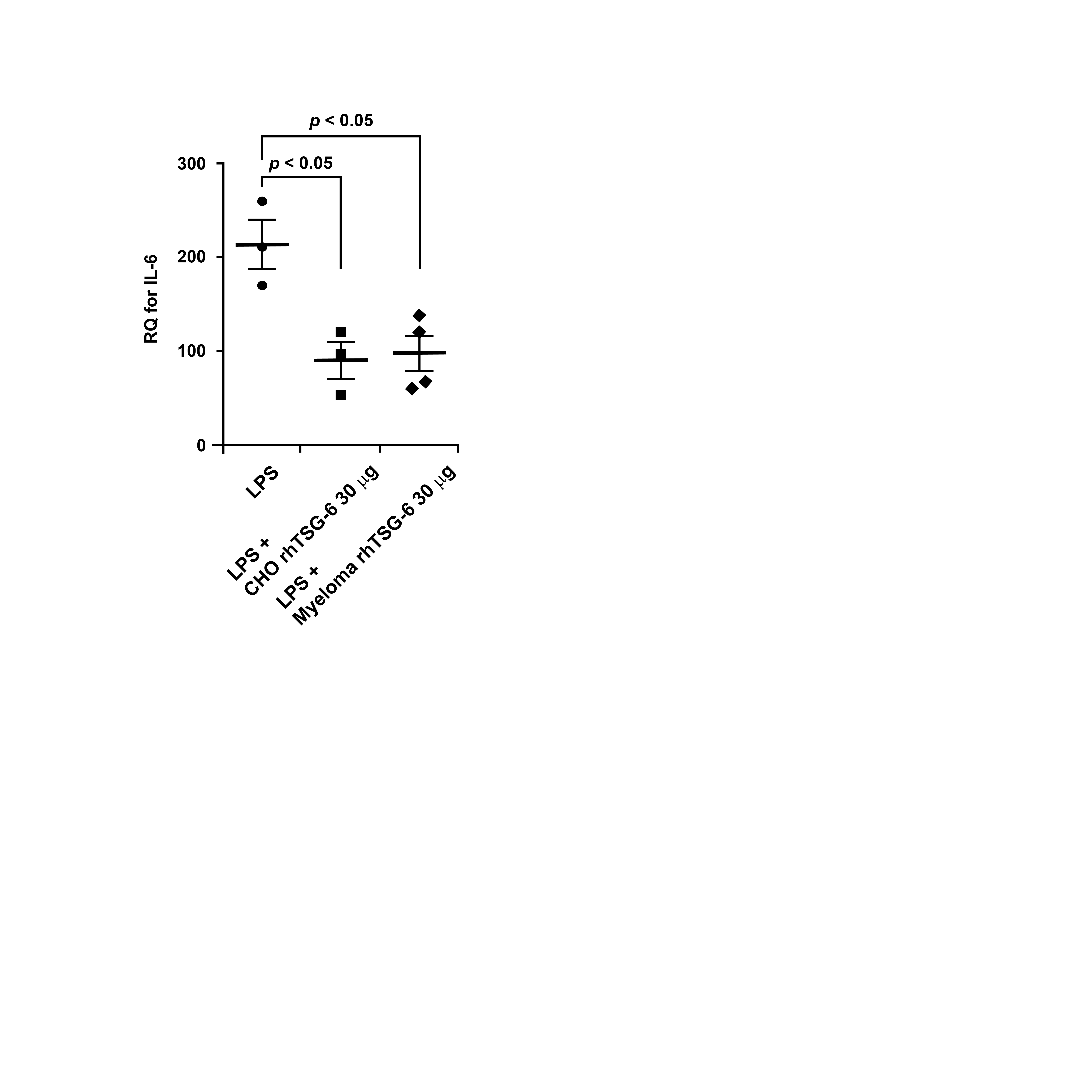

Supplement: S6 Fig — LPS (30 μg) was injected into a tail vein of BALB/C followed immediately by injection of rhTSG-6. Mice were killed 6 h later, spleens harvested and spleens assayed by RT-PCR. (TIF) [file pone.0147553.s006.tif]
