# Supplementary material for: Scalable Production of a Multifunctional Protein (TSG-6) That Aggregates with Itself and the CHO Cells That Synthesize It
Source: PLoS One. 2016 Jan 21;11(1):e0147553. doi: 10.1371/journal.pone.0147553 (PMC4721919; doi:10.1371/journal.pone.0147553)
Supplement: S1 Table — (DOCX) [file pone.0147553.s007.docx]

S1 Table. Purification process of rhTSG-6 proteins form 5 liter of CHO cell cultured media.

| Steps | Buffers | Buffer column volumes |
| --- | --- | --- |
| **1. Ni-Sepharose Excel resin**:  Column equilibration | 20 mM Phosphate buffer [pH 7.4] 500 mM NaCl 2 M Urea 0.1 % Triton-100 | 10 |
| 1-1. 1^st^ Wash :   Endotoxin removal | 20 mM Phosphate buffer [pH 7.4] 500 mM NaCl 2 M Urea 0.025% Triton-114 0.1% Tween 20 20 mM Imidazole [pH 7.0] | 25 |
| 1-2. 2^nd^ Wash :   Detergent removal | 20 mM Phosphate buffer [pH 7.4] 500 mM NaCl 2 M Urea 10 mM Imidazole [pH 7.0] | 40 |
| 1-3. Elution | 20 mM Phosphate buffer [pH 7.4] 500 mM NaCl 2 M Urea 500 mM Imidazole [pH 7.0] | 4 |
| 1-4. Dialysis | 50 mM Tris-HCl [pH 8.0] 50 mM NaCl 2 M Urea |  |
| **2. Q Sepharose FF resin**:  Column equilibration | 50 mM Tris-HCl [pH 8.0] 50 mM NaCl 2 M Urea | 10 |
| 2-1. Wash | 50 mM Tris-HCl [pH 8.0] 150 mM NaCl 2 M Urea | 20 |
| 2-2. Elution | 50 mM Tris-HCl [pH 8.0] 400 mM NaCl 2 M Urea | 3 |
| **3. Dialysis** | PBS |  |

Note:
1. Every procedure at room temperature.
2. Ni-Sepharose Excel resin bed volume: 70 ml
3. A-Sepharose FF resin bed volume: 100 ml
4. Flow rate for both columns: 3 ml/min
